# New Appliances and Things Medical

**Published:** 1894-10-06

**Authors:** 


### NEW APPLIANCES AND THINGS MEDICAL.

[All preparations, appliances, novelties, &c., of which a notice is desired, should be sent for the Editor, to care of The Manager, 428, Strand, London, W.C.]

### IRON JELLOIDS.

(Warrick Brothers, 18, Old Swan Lane.)

This new form, for the administration of the pill Mass. which goes by the name of Bland's pill, is a real advance in pharmacy, and deserves a wide popularity. The manufacturers have found that by mixing with the pill mass a small quantity of specially prepared jujube material, that the action of the air on the iron is prevented, and that the latter is preserved in the assimilable form of sub-carbonate. Thus the first advantage to be noted is that the iron jelloids will keep, a property which is not possessed by the ordinary form of Bland's pill. The second advantage that the jelloids possess is that they are easily swallowed and delicately flavoured. This is an exceedingly important point, for in the vast majority of cases in which Bland's pill is prescribed, the patient is a young and anæmic girl, in whom hysterical symptoms are often present, and who cannot or will not swallow anything in the form of a pill. In a few cases of this description we have recommended the iron jelloids, and have been surprised at the ease with which they have been swallowed. We can confidently recommend the iron jelloids as a most valuable substitute for the ordinary form of Bland's pill.

### S. KUTNOW AND CO.

(66, Holborn Viaduct.)

This firm have sent us samples of various well-made and useful articles. First, their improved effervescent Carlsbad powder. This powder, though containing all the essentials of the evaporated Carlsbad salts, has been made palatable and refreshing by the addition of the effervescing material, and thus enables them to be taken with comfort, which the natural salts certainly cannot be. It is well known that crude Carlsbad salts are very nauseous, although they are of great use in liver, gall bladder, and various gouty conditions. Messrs. Kutnow and Co.'s Carlsbad powders, whilst preserving all the medicinal properties of the crude salts, possess none of those objectionable features, and so enable the morning dose to be taken, not only without a wry face, but with possible enjoyment and undoubted satisfaction. The effervescent salts in smaller doses are also an excellent aperient for women and children. Kutnow's anti-asthmatic cigarettes and powder are both good. We gave the samples sent to an asthmatic patient, who was soon eloquent in their praise. We are always glad to find a new brand of anti-asthmatic cigarettes, as the disease is one which requires the remedy to be changed occasionally. Our patient's report being so good, and the vapour of both remedies being less stifling than many similar compounds, we can speak with confidence of their efficacy, and strongly recommend their extensive use.

### NEW "BACKED," LINT.

Messrs. T. Christy and Co., 25, Lime Street, E.C., have sent us a sample of Christia Backed Lint. It is claimed for the backing that it is hot and cold water proof, also spirit, chloroform, oil, and grease proof. We have tried it with the various agents mentioned, and find these claims to be fully justified in practice. Chloroform has a very slight effect upon it, but chloroform will not pentrate this lint when it is poured into a cup of this material. It is a most excellent preparation, and likely to be useful in a number of ways where an absorbent non-penetrable material is wanted for the application of liniments or other fluids or semi-fluids without the risk of soiling the clothing, and where also the question of cost must come in, as in those cases when spongiopiline is required. This latter is so dear that only where "expense is of no consequence" can it be used freely, whereas the small cost of the new backed lint places it within the means of everybody. It deserves on its merits to become popular with the profession, and to command an extensive sale.

